# Role of the Extracellular Traps in Central Nervous System

**DOI:** 10.3389/fimmu.2021.783882

**Published:** 2021-11-16

**Authors:** Xinyan Wu, Hanhai Zeng, Lingxin Cai, Gao Chen

**Affiliations:** Department of Neurological Surgery The Second Affiliated Hospital, Zhejiang University School of Medicine, Hangzhou, China

**Keywords:** extracellular traps, neuroinflammation, blood–brain barrier (BBB), stroke, neurodegenaration, CNS

## Abstract

It has been reported that several immune cells can release chromatin and granular proteins into extracellular space in response to the stimulation, forming extracellular traps (ETs). The cells involved in the extracellular trap formation are recognized including neutropils, macrophages, basophils, eosinophils, and mast cells. With the development of research related to central nervous system, the role of ETs has been valued in neuroinflammation, blood–brain barrier, and other fields. Meanwhile, it has been found that microglial cells as the resident immune cells of the central nervous system can also release ETs, updating the original understanding. This review aims to clarify the role of the ETs in the central nervous system, especially in neuroinflammation and blood–brain barrier.

## Introduction

**Figure 1:**
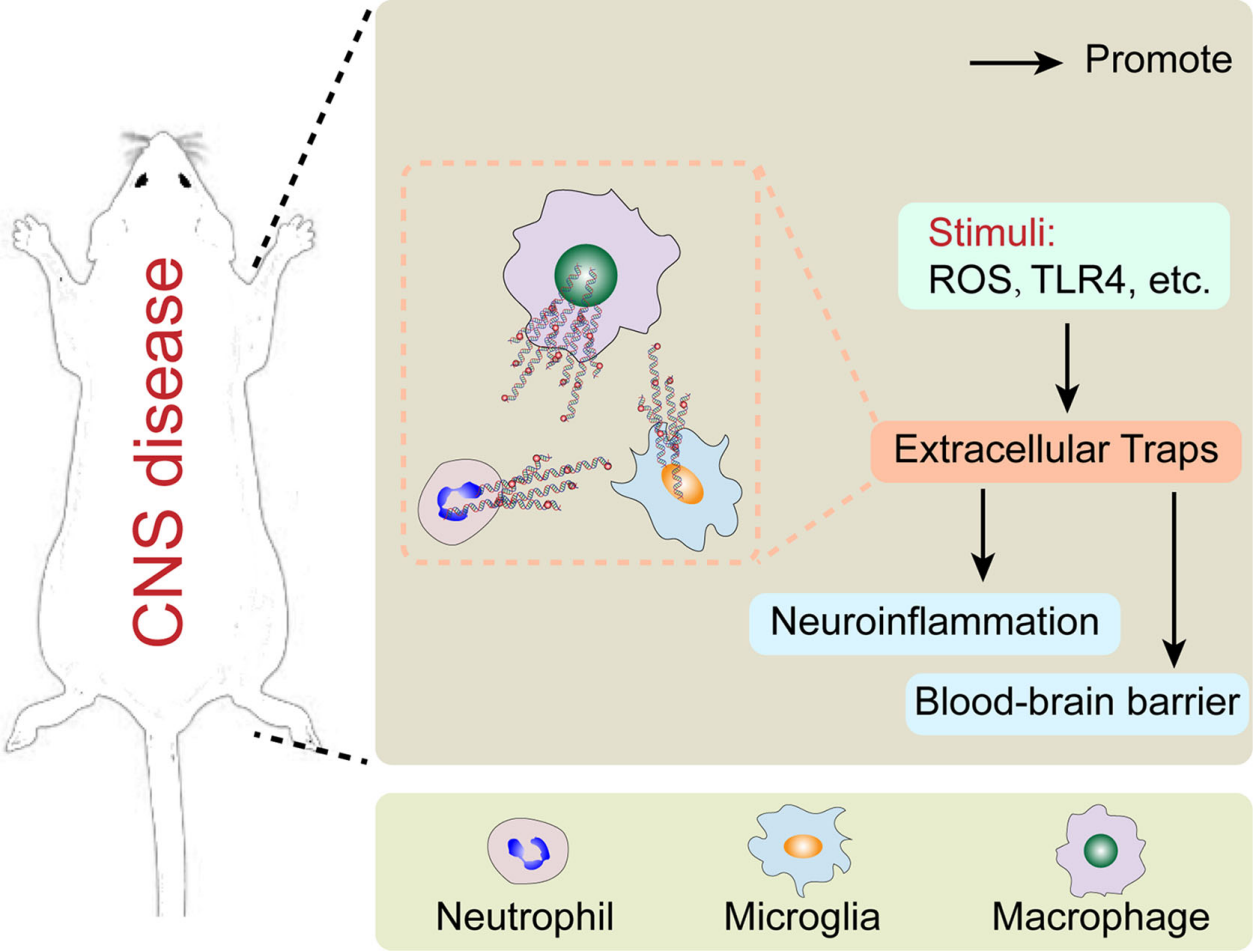
The major cells forming extracellular traps in central nervous system disease and their potential mechanism.

Extracellular traps (ETs) were first found in neutrophils and regarded as a host defense in response to bactericidal proteins and peptides (1). ETs were also considered to play a role in the inappropriate clearance of dead and dying cells in systemic lupus erythematosus (SLE) (2). They existed in a polynucleosome form, where histones associated with DNA tightly (3). Neutrophil extracellular traps (NETs) are large, extracellular, web-like structures of cytoplasmic and granular proteins clustered on a scaffold of decoagulated chromatin (1). Nuleus is the majority origination of the NET DNA, but mitochondrial DNA is also involved in the composition of NETs (1). According to the current research, the role of NETs is not limited to preventing microbial invasion but also participation in immune-related diseases (1, 4–8). With the development of research, more cells have been found to be involved in the formation of ETs (**Figure 1**). The other leukocytes including mast cells, eosinophils, and basophils also have been found to produce extracellular traps (9–13). The formation of ETs seems to play an alternative role in defense when the phagocytic capacity of cells is overtaxed (8). The characteristic of ETs is the DNA release related to histones and granule proteins, forming an extracellular web-like structure (14). This structure can capture and kill some microorganisms by acting as an immune defense (15). On the one hand, ETs can protect the body in response to the invasion of pathogenic microorganism. On the other hand, the excessive release of ETs can cause adverse effects in some diseases such as autoimmune diseases, cancers, and so on (16–21). Thus, the balance between the protective ET formation and the efficient elimination of excessive ETs still needs to be considered. Recently, the significant role of ETs in the central nervous system has come to be recognized in various related diseases. In this review, we described the development trend of ETs and the crosstalk between ETs and peripheral or central immune system.

## NETs

**Figure 2:**
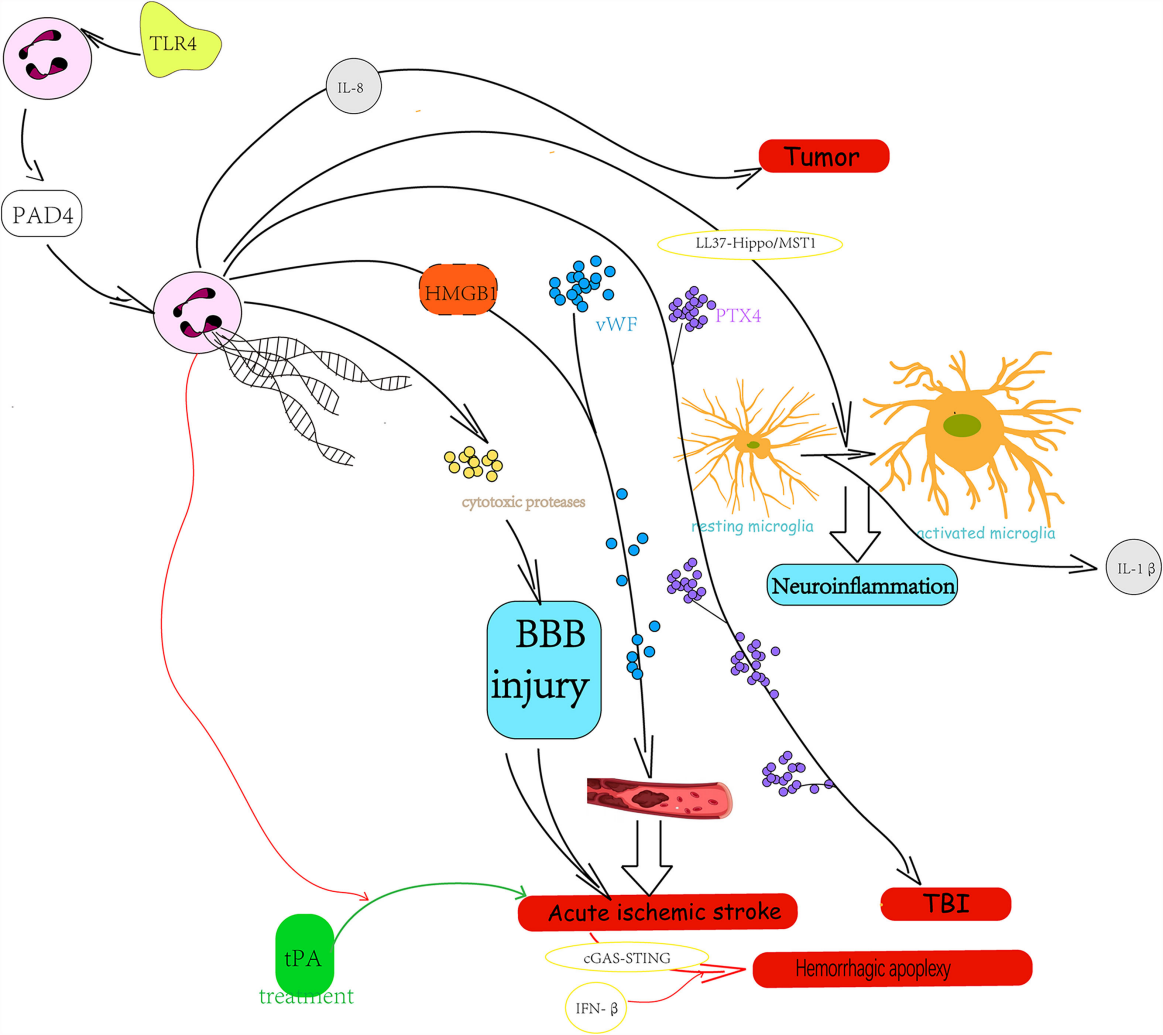
The role and possible mechanism of neutrophil extracellular traps in central nervous system disease.

Neutrophils have specialized in the formation of NETs, which are the most studied type of extracellular traps. As mentioned above, NETs can fight against microbes through immobilizing function and their antimicrobial compound equipment, which means the physiological functions of NETs include immune defense and autoimmunity. Moreover, the evidence for the inflammation-regulation action of NETs has been accumulating (22, 23). It has shown that the higher density of NET effect is a double-edged sword: on one hand, aggregated NETs can isolate the blocks of materials with immunostimulatory activity, leading to the limitation of immune reactivity and inflammation to sterile agents (22, 24, 25). On the other hand, tissue damaging is also one of the properties of NETs, suggesting that the higher density of NETs can result in an enlarged injury to tissue (25–27). The existing studies have highly assumed that neutrophil aggregation and NET formation might be interdependent, but the specific relationship and mechanisms between them remain unclear. As crucial cells of innate immunity, neutrophils are seldom found in the central nervous system (CNS) under normal conditions because of the presence of the blood–brain barrier (BBB). However, neutrophils can be activated in response to CNS diseases or exogenous stimulus and then injure the BBB. Meanwhile, the infiltration of neutrophils and the release of NETs increase. This phenomenon has been reported to exist in various neurological diseases, such as stroke, traumatic brain injury (TBI), neurodegenerative diseases, autoimmune diseases, and tumors. In related diseases, NETs always play a role of aggravating diseases through its properties and damaging the integrity of BBB (**Figure 2**). The following are classified according to different types of diseases and will show how the neuroinflammation and BBB damage caused by NETs influence the concrete neurological diseases.

## Acute Ischemic Stroke

Acute ischemic stroke is a hypoxic-ischemic disorder associated with a sterile inflammatory reaction (28), promoting immune cell migration and infiltration to the brain parenchyma (29, 30). Animal studies showed that neutrophils infiltrated the ischemic areas of the brain within a few hours after the onset of experimental ischemia, and Perez-Puig et al. described the presence of citrullinated histone 3, a hallmark of NET formation, in the ischemic brain after 24-h ischemia (31). Peña-Martínez et al. indicated that the neutrophil activation by platelet Toll-like receptor 4 (TLR4) could result in NETosis and NETs could act as assembly platforms for atherothrombosis by binding platelet-derived microparticles (PMPs) and clotting factors (32–34). Recent research considered that NETs promoted vaso-occlusion, and this process was initiated through von Willebrand factor (vWF) (35, 36). Meanwhile, it has been reported that the outer shell of thrombus samples from clinical acute ischemic stroke (AIS) patients acting as a protective barrier against thrombolysis are composed of fibrin, RBC, vWF, leukocytes, and nucleated cells (37–41), which suggest the potential value of therapy targeting NETs in thrombolysis. Additionally, DNAse 1 has been shown to target NETs and extracellular DNA in thrombi retrieved from patients with AIS and then accelerates *ex vivo* lysis of cerebral thrombi (36, 42, 43).

The existing research indicated that NETs play a role in neurological dysfunction after ischemia probably through the BBB destruction (31, 44). It has been reported that neovascularization and perfusion of the vascular structure in the peri-ischemic brain have important roles in stroke recovery (45, 46), while NETs can release many cytotoxic proteases such as histone, elastase, and MPO, which directly induce endothelial cell damage to increase vascular permeability and then break BBB (44). Kim et al. demonstrated that high-mobility group box-1 (HMGB1), a prototypic danger-associated molecular pattern (DAMP), is involved in NET-mediated neuronal damage in the ischemic brain, where disulfide HMGB1 can induce NETosis *via* CXCR4 and TLR4 (47, 48). The experiments by Kim et al. are also the first to describe the temporal and spatial progressions of NETosis after middle cerebral artery occlusion (MCAO) using an intraluminal model and report that the main route of neutrophil infiltration from peripheral blood vessels after brain ischemia follows the route: leptomeningeal vessel → Virchow-Robin space → perivascular space → brain parenchyma (47).

Although the thrombolysis with tissue plasminogen activator (tPA) is the only approved pharmacological therapy for acute ischemic stroke, it presents a major clinical problem as an increased risk of intracerebral hemorrhage (49–51). Ranran et al. also revealed that tPA treatment in mice with thrombotic stroke increased NET formation and that activation of the cGAS-STING pathway and production of IFN-β participated in NET-mediated effects on tPA-associated cerebrovascular complications in stroke (52). These NETs were related to the increased risk of intracerebral hemorrhage, all of which could be attenuated by clearing NETs with DNase I or inhibiting NET production by PAD4 deficiency (52). The series of research results mean the potential therapeutic value of targeting NETs in both thrombolysis and the adjuvant therapy of tPA.

Meanwhile, the present studies have shown that neutrophil influx is more prominent after permanent than after transient MCAO and the frequency and intensity of NETosis are significantly greater after permanent MCAO, which suggests a relationship between NETosis and disease severity (31, 47, 53). Moreover, it was observed that NET marker levels were associated with stroke severity at onset and discharge from hospital as evaluated using NIHSS and mRs scores in the plasma of acute ischemic stroke patients, and that increasing levels of CitH3 at onset were associated with all-cause mortality at 1-year follow-up visits (54). Therefore, NETs may be a useful prognostic maker in acute ischemic stroke in the future according to the current research results.

## Hemorrhagic Apoplexy

The proportion of nontraumatic intracerebral hemorrhage (ICH) in acute strokes is ~10%–15%, and nontraumatic ICH has a much higher risk of mortality than ischemic strokes or subarachnoid hemorrhage (55, 56). A recent research reported the NET infiltration in the brain of patients who died from spontaneous intracerebral hemorrhage (sICH), suggesting that NETs might interact with early hemostasis within the hematoma core and with the surrounding neuroinflammatory response (57). Orbán-Kálmándi et al. pointed out that a modified clot lysis assay, incorporating the effect of NETs, could suggest unfavorable outcomes in spontaneous, nontraumatic ICH based on a prospective observational study data (58). Also, in subarachnoid hemorrhage (SAH), the current studies revealed that NETs can induce the alteration of microglia into a proinflammation subtype in order to promote neuroinflammation and cause adverse consequences, suggesting that NETs may be a potential target for the treatment SAH in its early phase (59, 60). According to available research, we can make a bold assumption that NETs may be a potentially effective therapeutic target to hemorrhagic apoplexy.

## Traumatic Brain Injury

TBI is a major public health issue, which may contribute to elevated intracranial pressure (ICP) and lead to neurological deterioration. A research identified NETs as the distinct mediators of cerebral edema which caused elevated ICP and neurological deterioration (61). The researchers revealed that the formation of NETs was induced by the activation of TLR4 *via* a PAD4-dependent mechanism and then resulted in neurological deficits (61). In 2021, researchers have demonstrated the presence of NETs in paraventricular nucleus (PVN) after TBI and NETs activated microglia dependent on the LL37-Hippo/MST1 pathway to facilitate the IL-1β release, which may lead to the occurrence of sympathetic excitation as a result (62). Oggioni et al. reported the presence of PTX3 in the mouse brain parenchyma, next to astrocytes, neurons, microglia, and endothelial cells, in the subacute phase of TBI for the first time (63). In view of the complex functions of PTX3 in different cells (64–67), this research considered that PTX3 may alleviate subacute pathological sequelae after TBI (63). Meanwhile, PTX3 is complexed with the NET components, which suggest that NETs may participate in the subacute phase of TBI through PTX3.

## Neurodegenerative Diseases

Alzheimer’s disease (AD) is one of the neurodegenerative disorders characterized by the progressive deterioration of cognitive functions. Its neuropathological features include amyloid-β (Aβ) accumulation, the formation of neurofibrillary tangles, and the loss of neurons and synapses (68). Neuroinflammation is a well-established feature of AD pathogenesis, and recent studies have identified several inflammation pathway genes associated with the risk of AD (69, 70). AD is also characterized by the loss of BBB integrity, which disrupts the clearance of Aβ and thus promotes Aβ accumulation in the brain, leading to neuronal injury and cognitive decline (71). Recent research have indicated that both the intravascular NETs and intraparenchymal NETs have an impact on AD (72). Zenaro et al. reported the existence of neutrophil–microglia crosstalk and intravascular and intraparenchymal NETs in AD in 2015 (73). This existence suggested that NETs can possibly damage the BBB and neurons in AD (73, 74). Additionally, the research supported the neutrophil-dependent brain damage in AD, showing that the migration of neutrophils produced IL-17 which has toxic effects on neurons directly and may recruit more neutrophils (73). In an AD mouse model, it has been found that the block of LFA-1 integrin can reduce the neutrophil adhesion in the brain microvasculature, alleviating the cognitive deficits (73). Interestingly, previous studies reported that the brain vasculature in AD humans produced more cytokines such as TNF-α, IL-1β, IL-8, and thrombin which triggered the intravascular NETs compared with the age-matched controls (75–80). A recent research has reported the presence of three times excessive level of NET formation in the peripheral blood of mild cognitive impairment (MCI) patients who are the precursor of AD and a positive correlation between the excessive level of NET formation and the content of Aβ (81). *In vitro* studies based on brain endothelial cells revealed increasing expression of cytokine genes which induce NETosis in response to the exposure to Aβ peptides (82). Moreover, Aβ peptides can further promote intravascular NETosis by inducing the generation of ROS and secreting more proinflammatory cytokines (72). Neutrophils invaded the brain parenchyma at the early stage of AD in mice model and produced NETs, leading to memory deficit (73). Similarly, the release of intraparenchymal NETs can also be influenced by related cytokines as intravascular NETs in the case of Aβ peptide exposure (73, 83). Meanwhile, a study pointed out that the intraparenchymal release of NETs can also respond to other fibrillary form of amyloids (84). The zurophilic granules of neutrophils release MMPs and serine proteases which can cause tissue injury and exacerbate the inflammatory response during the generation of NETs. MMPs activated at the stimulation of neutrophils are involved in the proteolysis of the extracellular matrix in order to cause damage to the brain parenchyma (85). The serine proteases such as NE can also degrade tissues not only by cleaving extracellular matrix proteins but also by inactivating the endogenous tissue inhibitors of MMPs (TIMPs) which can also be inhibited by MPO localized within NETs (86, 87). The existing research suggests that NETs seem to be a new therapeutic target to ease the AD progression, and more research is still needed to investigate the mechanism of NETs in AD (88).

In addition to AD, Parkinson’s disease is also a kind of neurodegenerative disorder resulted from the accumulation of amyloid fibrils formed by specific misfolded proteins (89). And in amyloid diseases, researchers observed that amyloid fibrils induced NETs release dependent on the NADPH oxidase system to a large extent (84). Although there are few studies focusing on the other neurodegenerative disorders, the study based on related pathomechanism has been proceeded, suggesting the potential therapeutic value of NETs in neurodegenerative disorders.

## Autoimmune Diseases

NETs were considered to contribute to the autoimmune diseases as early as the phenomenon of NET release was first reported (1). Current researches have shown that NETs are closely related to a of lot of autoimmune diseases including those that may be affecting the central and peripheral nervous systems (90–92). Multiple sclerosis (MS) is a chronic inflammatory, demyelinating disease, and the mechanism of MS includes both a complex genetic trait and environmental factors (93). As mentioned in a recent review, NETs may have a cytotoxic effect on the BBB and facilitate the damage of adjacent neurons and other cells of the CNS in MS (94). Meanwhile, the NET-associated decrease of proteins can ease the MS progression and increase the BBB integrity (94, 95). Interestingly, the NETs in the serum of MS patients are elevated compared with the controls, and there is a partial sex difference in the extent of elevation of NETs (92, 95, 96). In mice model, Allen revealed that murine neutrophil metastases through activated cerebrovascular endothelial cells could induce a proinflammatory and neurotoxic phenotype and lead to the release of NET as a result (74). Although researchers have indicated the NET-related potentiation of proteases to modify the immune complexes, the real mechanism in human MS is still unclear (97).

In addition to MS, NETs are also involved in other neuropsychiatric symptom. According to a recent review, it concludes a hypothesis for the cognitive dysfunction in systemic lupus erythematosus (SLE): MMP-9 released by prestimulated LDGs can degrade the basal lamina and damage the integrity of BBB. And then the anti-NR2A/B antibodies further activate the BBB, increasing the expression of endothelial cell adhesion molecules. Afterwards, the neutrophils recruit, roll, adhere and transmigrate, leading to further NETosis. Finally, the NETs release leads to neurotoxity through inducing neuron death, causing the neuropsychiatric manifestations of SLE as a result (98). In microscopic polyangiitis (MPA), it is considered that NETs may be involved in the pathogenesis of neuropathy and suggests the therapeutic strategies targeting NETs based on the nerve biopsy samples from MPA patients (99).

## Malignant Brain Tumors

Malignant brain tumors can be both primary tumors originated in the brain such as gliomas and exogenous tumors that metastasize into the brain such as nonsmall-cell lung carcinoma (NSCLC) (100). The prognosis for both primary and metastatic brain malignancies is poor, mainly due to the limitations of standard treatments, thus researchers turn to the tumor microenvironment (101). Based on the previous studies, NETs seemed to facilitate the cytotoxic effect and inhibit the spread of cancer cells as a result of inducing epithelial and endothelia cell death (26, 102). On the contrary, neutrophils have been proved to facilitate the metastasis of tumor in experiments and animal studies in different cancers (103–107). In 2013, Cools-Lartigue et al. pointed out that the NETs can promote tumor metastasis through isolating circulating tumor cells (108). Moreover, the recent research reported that NETs formed during LPS- or tobacco smoke-induced lung inflammation can awaken dormant cancer cells and cause metastasis in mice dependent on FAK/ERK/MLCK/YAP pathway (109–111). In glioma cells, it has been considered that NETs could induce the expression of IL-8 which is correlated with tumor burden and prognosis through a HMGB1- and RAGE/ERK/NF-κB axis-dependent manner (112). Furthermore, the IL-8 produced by glioma can cause in turn the formation of NETs (112). T-cell immunoglobulin and mucin domain-3 (TIM-3) also has been proven to interact with HMBG1 in TADCs and then preventing the nucleic acids from localizing into the endosomal vesicles, thus playing a role in blowing the antitumor effect of tumor-associated dendritic cells (TADCs) (113–115). Additionally, the administration of anti-Tim-3 mAb during chemotherapy has been demonstrated to lead to tumor regression (113). Interestingly, a current research has reported that TIM-3 can suppress the uptake of extracellular DNA in intratumoral dendritic cells in order to limit the activation of cGAS-STING pathway, which may influence the production of NETs (116). While these discoveries unstated the type of tumor and the specific role of NETs in the referred mechanism, they still suggest the future research interests of NETs in malignant brain tumors relating to HMBG1-TIM-3. Toll-like receptor 2 (TLR2), one of the HMBG1 receptors, has been considered to involve in the production of NETs, and HMGB1-mediated TLR2 signaling plays a critical role in eliciting glioblastoma regression, suggesting the prospect of NETs in malignant brain tumors (117–119). Although the current research has demonstrated the role of neutrophils in glioma (107), further studies are still needed to clarify the protumor and antitumor functions of NETs in glioma.

## METs

Macrophages comprise a diverse group of cells and demonstrate remarkably various functions. Macrophage functions ranged from supporting development, maintaining homeostasis, keeping immune surveillance, and regulating tissue remodeling and repair (120). The formation of METs was initially found at the stimulation of *Mycobacterium tuberculosis* in 2013 (121). Although the following research focused on the formation of METs *in vitro* and *in vivo*, the mechanism of the formation of METs is still unknown. It has been reported that microorganisms such as *Toxoplasma gondii*, *Candida albicans*, *Staphylococcus aureus*, *Haemophilus influenza*, *Klebsiella pneumoniae*, and *Escherichia coli* can stimulate the formation of METs (122), but METs play opposite roles in different infections caused by diverse microorganisms (123–125). Pertiwi et al. reported that METs were significantly more numerous in the late stages of thrombus formation and were mostly located around and inside the lipid core, suggesting that METs involved in the thrombus formation. Although the specific role of METs in thrombus formation is unclear, this finding supports the involution of METs in noninfectious diseases (126). While the studies on the role of METs in CNS are few, the research focusing on the crosstalk between monocyte-macrophage system and CNS is not in the minority, including its effect on glial cells. Therefore, the research based on the role of METs in CNS will be more in the foreseeable future, which is supposed to become a new target.

## MiETs

Microglia are a resident mononuclear phagocyte population in the CNS and are gatekeepers of CNS immunology, involved in the CNS maintenance (127, 128). A previous study has reported that microglia can release extracellular traps (MiETs) in response to the *Listeria* infection *in vivo* and *in vitro* (129). Then, a following research found that MiETs could be induced by dopamine *in vitro* and interestingly the formation of MiETs did not lead to immediate cell death (130). Although the mechanism of the formation of MiETs is unclear, the existing results suggest that the role of MiETs in CNS steady state including infection, neuroinflammation, and glioma are intriguing areas for future investigations. As one of the most important immune cells in CNS, the research of microglia has been continued endlessly. The interaction between microglia and astrocytes has been well verified in CNS diseases such as vascular, tumor, and trauma. As a result, as one of the rich mineral cells, it is of great value to continue to be explored to reveal its potential pathogenesis.

## Conclusion

The increasing evidence shows that the presence of ETs in CNS plays different roles. In this review, we described the roles of ETs in different diseases, especially focusing on the integrity of BBB and neuroinflammation. According to a lot of existing research focusing on NETs, we have demonstrated that the excessive release of NETs involves in the breakdown of BBB integrity and facilitating the neuroinflammation through releasing metalloproteinases, proteases, cytokines, extracellular histones, DNA, and ROS. Furthermore, more research is demanded to be implemented to focus on the other roles of ETs in the neuroinflammation and BBB integrity. While the relating mechanism is still unclear, the current results suggest that ETs may become the potential therapeutic targets for CNS diseases to improve prognosis.

## Author Contributions

LC has organized manuscripts. All authors contributed to the article and approved the submitted version.

## Funding

This work was supported by the National Key R&D program of China (2018YFC1312600, 2018YFC1312603), the National Natural Science Foundation of China (Nos. 81771246, 81971099, and 81870908), TCM Science and Technology Plan of Zhejiang province (2017ZZ013), TCM Key Discipline of Zhejiang province (2017-XK-A39), and the Natural Science Foundation of Zhejiang Province (LY19H090019).

## Conflict of Interest

The authors declare that the research was conducted in the absence of any commercial or financial relationships that could be construed as a potential conflict of interest.

## Publisher’s Note

All claims expressed in this article are solely those of the authors and do not necessarily represent those of their affiliated organizations, or those of the publisher, the editors and the reviewers. Any product that may be evaluated in this article, or claim that may be made by its manufacturer, is not guaranteed or endorsed by the publisher.
